# Insight into the Formation of Winter Black Carbon and Brown Carbon over Xi’an in Northwestern China

**DOI:** 10.3390/toxics14010093

**Published:** 2026-01-20

**Authors:** Dan Li, Qian Zhang, Ziqi Meng, Hongmei Xu, Peng Wei, Yu Wang, Zhenxing Shen

**Affiliations:** 1 Department of Environmental Science and Engineering, Xi’an Jiaotong University, Xi’an 710049, China; lidan@ieecas.cn (D.L.); xuhongmei@xjtu.edu.cn (H.X.); zxshen@mail.xjtu.edu.cn (Z.S.); 2 State Key Laboratory of Loess Science, Institute of Earth Environment, Chinese Academy of Sciences, Xi’an 710061, China; 3 Key Laboratory of Northwest Resource, Environment and Ecology, MOE, Xi’an University of Architecture and Technology, Xi’an 710055, China; mengziqi@xauat.edu.cn (Z.M.); 15943255731@163.com (Y.W.); 4 Division of Environment and Sustainability, The Hong Kong University of Science and Technology, Hong Kong, China; pengwei@sdnu.edu.cn

**Keywords:** black carbon, brown carbon, winter periods, source identification, GAM model

## Abstract

This study evaluates the effectiveness of air pollution control measures in Xi’an, China, by investigating long-term changes in the concentrations, optical properties, and sources of black carbon (BC) and brown carbon (BrC). Wintertime observations of PM_2.5_ carbonaceous aerosols were conducted over multiple years using a continuous Aethalometer. The data were analyzed using advanced aethalometer models, potential source contribution function (PSCF) analysis, and generalized additive models (GAMs) to deconstruct emission sources and formation pathways. Our results revealed a significant decrease in the mass concentration and light absorption coefficient of BC (b_abs_-BC) between the earlier and later study periods, indicating successful emission reductions. In contrast, the light absorption from BrC (b_abs_-BrC) remained relatively stable, suggesting persistent and distinct emission sources. Source apportionment analysis demonstrated a temporal shift in dominant regional influences, from biomass burning in the initial years to coal combustion in later years. In addition, GAMs showed that the primary driver for liquid fuel-derived BC transitioned from gasoline to diesel vehicle emissions. For solid fuels, residential coal combustion consistently contributed over 50% of BC, highlighting that improvements in coal combustion technology were effective in reducing BC emissions. Furthermore, a substantial fraction of BrC was increased, with nocturnal peaks associated with high relative humidity, emphasizing the aqueous-phase formation influences. Collectively, these findings demonstrated that although certain control strategies successfully mitigated BC, the persistent challenge of BrC pollution necessitates targeted measures addressing secondary formation and primary fossil fuel sources.

## 1. Introduction

Carbonaceous aerosols that absorb light influence the climate by enhancing surface heating, promoting evaporation, and modifying cloud properties through direct and semi-direct radiative effects [1,2,3,4]. These light-absorbing particles are generally classified as black carbon (BC), which strongly absorbs across the visible spectrum, and brown carbon (BrC), which predominantly absorbs in the ultraviolet (UV) and short-visible wavelengths [5,6,7].

The recent studies have found that under certain regional conditions, energy-related incomplete combustion remains one of the primary sources of BC emissions, including the burning of fossil fuels, transportation fuels, and biomass. In China, BC does great harm to regional air quality [8,9]. Lu et al. [10] indicated that by 2030, China’s BC emissions may reach 166 Tg, and with the current energy mix. Moreover, Yu et al. [3] confirmed that BC emitted from different sources differs considerably in absorbance and radiation contribution. For example, an observation-constrained study in Nanjing found that BrC absorption accounted for 8.7–34.1% of total aerosol absorption at 370 nm, and that BrC’s average instantaneous direct radiative forcing was ~15% of that contributed by BC [11]. In parallel, measurements of residential biomass combustion emissions in Europe show BrC’s contribution to particulate light absorption varied between ~1% and 21% across 370–950 nm [12]. However, substantial uncertainties persist in quantifying BrC’s radiative impact—especially due to its heterogeneous composition and optical properties. The variability in imaginary refractive index, atmospheric aging, photobleaching effects, and source-dependent chromophore distributions all limit robust estimation of BrC’s global forcing [13]. In contrast to the pure light-absorbing substance BC, BrC is defined as a compound with a complex chemical composition, wide sources, and strong atmospheric reactivity. Moreover, BrC is composed of both primary and secondary organic carbons, which are often associated with various anthropogenic activities as well as complicated atmospheric processes.

Xi’an is located in central-northwest China, which is situated in the center of the Weihe Plain surrounded by Mt. Qinling and the Loess Plateau. Frequent occurrences of winter haze pollution have caused widespread public concern in this region, particularly during the winter of 2013 [14,15,16]. Since 2013, a series of air pollution control measures such as vehicle license plate number restrictions, “coal-to-gas”, and “clean-coal combustion” have gradually been implemented through government policy. After continual efforts, the occurrence of haze days has been considerably reduced over the past six years. In winter, traffic control and central heating activities have changed the air quality significantly over the years. Overall, comparing the levels, optical properties, sources, and regional transport of BC and BrC is essential to evaluating the effectiveness of air quality management strategies. Therefore, based on datasets of 5 min measurements over Xi’an, this study was conducted to (1) examine the 3-year variations in winter BC and BrC levels, sources, optical properties, and potential regional transport contribution in this region; (2) classify BC and BrC species, including liquid source- and solid source-derived BC and primary and secondary BrC, and assess their distribution differences during these years; and (3) quantify changes in multiple influential factors for different BC and BrC species, including meteorological indices and gaseous pollutants, before and after air pollution control measures.

## 2. Materials and Methods

### 2.1. Measurement and Classification of BC and BrC

**Figure 1:**
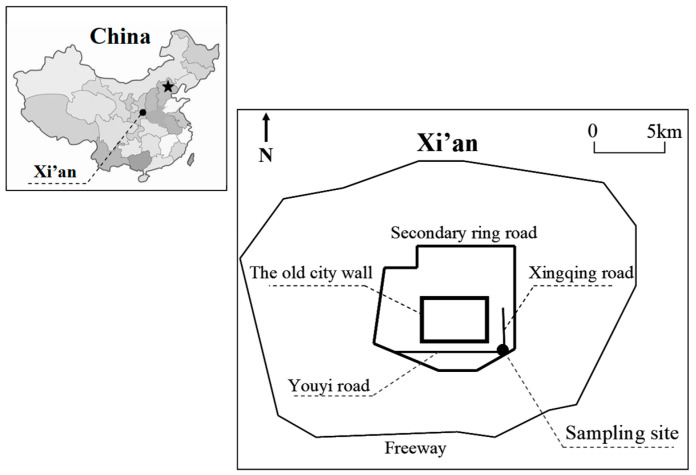
Location of sampling site.

Figure 1 shows that the sampling site is situated on the roof (~15 m high) of the laboratory building at Xi’an Jiaotong University in southeastern Xi’an city. The site is surrounded by residential areas, campus, commercial streets, and the south second ring and Xingqing roads [17,18,19]. The aerosol light absorption coefficient (b_abs_, Mm^−1^) was determined by monitoring the continuous, real-time reduction in light intensity as it passed through a glass fiber filter, where fine particulate matter (PM_2.5_) had been collected. Observations commenced in Xi’an in December 2013, utilizing a seven-wavelength Aethalometer (AE-31, Magee Scientific, Berkeley, CA, USA), operating at 370, 470, 520, 590, 660, 880, and 950 nm. In this study, a total of approximately 280,000 aerosol b_abs_ data points were collected during winter from 2013 to 2018, including December 2013–February 2014, December 2015–February 2016, and December 2017–February 2018. The inlet air flow of AE-31 was set as 5.0 L·min^−1^ and was calibrated before field measurements.

AE-31 b_abs_ data were post-processed with two corrections: a loading adjustment addressing nonlinear behavior as deposits accumulated on the filter, and a term accounting for scattering of light by the glass-fiber substrate [20,21]. Loading-related artifacts and filter-substrate scattering in the AE-31 were systematically characterized previously. This statement was originally made by Zhang et al. [22]. The derailed separating method of BC and BrC was presented by Olson et al. [23]. Therefore, BrC b_abs_ levels were calculated with two assumptions: The first assumption was that BC was the sole light absorber at 880 nm, and its absorption Ångstrom exponent (AAE) value was 1.0. The second assumption was that b_abs_ at 370 nm was mixed with BrC and BC. Subtracting the b_abs,_ the BC value from b_abs_-370 nm yielded the net BrC absorption coefficient. Details regarding the b_abs_-BrC calculations employed in this study were provided by Zhang et al. [24].

### 2.2. BC Source Identification Using the Aethalometer Model

Zhang et al. [25] identified two dominant sources of BC in China: liquid-fuel combustion (primarily vehicular) and solid-fuel burning (coal and biomass). At any selected wavelength, b_abs_ may be partitioned into liquid b_abs_-BC (b_abs_-_Liquid_) and solid b_abs_-BC (b_abs_-BC_Solid_). A straightforward separation procedure is adopted as follows:

(1) $$\frac{b_{\mathrm{abs}\;370}^{\mathrm{liquid}}}{b_{\mathrm{abs}\;880}^{\mathrm{liquid}}}={\left(\frac{370}{880}\right)}^{-({\mathrm{AAE}}^{\mathrm{liquid}})}$$

(2) $$\frac{b_{\mathrm{abs}\;370}^{\mathrm{solid}}}{b_{\mathrm{abs}\;880}^{\mathrm{solid}}}={\left(\frac{370}{880}\right)}^{-({\mathrm{AAE}}^{\mathrm{solid}})}$$

(3) $$b_{\mathrm{abs}\;880}=b_{\mathrm{abs}\;880}^{\mathrm{liquid}}+b_{\mathrm{abs}\;880}^{\mathrm{solid}}$$

(4) $${\mathrm{BC}}^{\mathrm{liquid}}=\frac{b_{\mathrm{abs}\;880}^{\mathrm{solid}}}{\sigma_{880}}$$

where b_abs 370_ and b_abs 880_ denote absorption at 370 and 880 nm, respectively; $b_{abs\lambda }^{liquid}$ marks the liquid-fuel share, and $b_{abs\lambda }^{solid}$ reflects the solid-fuel contribution. b_abs_ is reported in Mm^−1^ (or 10^−6^ m^−1^); AAE_liquid_ has a value of 1 for liquid fuel, and AAE_solid_ has a value of 2 for solid fuel [26]. Here, σ_880_ denotes the BC mass attenuation coefficient, 16.6 m^2^·g^−1^ at 880 nm.

### 2.3. Separation of Secondary BrC Absorption

BrC arises from primary as well as secondary pathways. Absorption by BrC_P_ reflects contributions from combustion-related sources together with non-combustion BrC. Primary BrC from non-combustion origins is chiefly biogenic and occurs mostly in the coarse mode, so for a PM_2.5_-focused analysis, it can be disregarded [27]. Accordingly, the non-combustion portion of BrC_P_ contributes negligibly to total b_abs_-BrC owing to its small mass within PM_2.5_. To quantify the remainder, we partitioned b_abs_ into a secondary component (b_abs_-BrC_S_) using a BC-tracer method. The resulting equations are provided below:

b_abs_-BrC_S_ = b_abs_-BrC − combustion b_abs_-BrC_P_ (5)

The primary combustion component of b_abs_-BrC is computed as

Combustion b_abs_-BrC_P_ = (b_abs_/BC) _P_ × [BC] (6)

where b_abs_-BrC denotes the total BrC absorption coefficient at wavelength λ; P indicates the primary fraction; and [BC] denotes the BC concentration at 880 nm, obtained via the procedure in Section 2.1. The term (b_abs_/BC)p, as defined per Equation (6), is the ratio between overall particle absorption and BC mass per volume for fresh combustion emissions and exhibits strong source sensitivity. Because this ratio is source dependent, selecting a representative value is nontrivial [28,29]. Consistent with our previous study [29], we estimated it using the minimum R-squared (MRS) criterion. This procedure generates multiple regressions relating BC to absorption by secondary BrC (refer to Figure S1 for R^2^). Assuming independence between BC and absorption by secondary BrC formed predominantly in ambient air, the chosen value of b_abs_-BrC_P_ is the one that yields the weakest association (is the smallest R^2^). After we obtained (b_abs_/BC)_p_ values at different wavelengths, we can then estimate the secondary BrC absorption during different sampling periods in Xi’an.

### 2.4. Potential Source Contribution Function (PSCF) and Cluster Analysis

PSCF was applied as an effective approach to identify potential source regions of b_abs_-BC and b_abs_-BrC across the sampling periods. Calculations were carried out in TrajStat, which relies on the MapWindow GIS ActiveX control open-source GIS framework (MapWindow open-source team, 2007) [30,31], with meteorological fields from the NCEP Global Data Assimilation System (GDAS). In addition, 24 h back trajectories ended in Xi’an (34.62° N, 108.93° E) at 500 m above ground level. The calculation was described as follows:

PSCF_ij_ = m_ij_/n_ij_ (7)

where m_ij_ and n_ij_ represent all endpoints and “high” endpoints falling within the ijth grid cell, respectively; the total amount of trajectory endpoints was calculated (the region of possible source was subdivided into i × j grid cells) at 0.5° latitude × 0.5° longitude in Xi’an. Thus, the calculated PSCF_ij_ was treated as a representative ratio of “high” grid points of b_abs_-BC and b_abs_-BrC events to their total grid points, respectively. These “high” events in this study were defined when the values of b_abs_-BC and b_abs_-BrC at the sampling site were larger than their own criterion values, respectively [32]. The criterion values were calculated as the 75th-percentile values of b_abs_-BC and b_abs_-BrC for the individual sampling periods [33,34].

Cluster analysis is a multivariate statistical technique to divide the total number of trajectory air masses into groups. For each twenty-hour trajectory, 24 x, y coordinates (i.e., trajectory location endpoints at each hour) were used as input variables for the clustering algorithm. A k-means clustering algorithm was used, and “Euclidean” was thus selected in TrajStat to evaluate the distances between latitudinal and longitudinal variables. Byčenkienė et al. [35] described this method in detail.

### 2.5. Generalized Additive Models (GAMs)

To assess the impact of control policies on BC and BrC, we applied generalized additive models (GAMs) to estimate how source-related pollutants and meteorology influence b_abs_-BC and b_abs_-BrC. The approach does not impose a fixed functional form on predictor–response relationships; instead, linear and nonlinear effects for each covariate are learned independently [36,37]. Predictors comprised meteorological variables, relative humidity, wind speed, and wind direction, and gaseous species SO_2_, NO_2_, and O_3_. We implemented four GAM specifications, given by

ln(b_abs_) = s1(SO_2_) + s2(NO_2_) + s3(O_3_) + s4(relative humidity) + te(U,V) + ε (8)

where b_abs_ denotes absorption coefficients for liquidBC, solidBC, BrC secondary, and BrC primary; si (i = 1, 2, 3, 4) are smooth functions; te is a tensor-product term; and ε denotes the GAM residual. Hourly meteorology was sourced from the national meteorological data portal of China, while SO_2_, NO_2_, and O_3_ were retrieved from the official site of the Xi’an Bureau of Environmental Protection. The five-minute b_abs_ series was aggregated to hourly means. Following [38], the combined effects of wind speed (m·s^−1^) and wind direction (rad) were examined via bivariate trend-surface modeling; formulations appear below:

U = wind speed × cos(wind direction)(9)

V = wind speed × sin(wind direction)(10)

where U > 0 denotes easterly flow, and V > 0 denotes northerly flow. Computations were performed in R (v. 3.6.1; R Foundation, Vienna, Austria), an open-source platform freely accessible online. GAMs were fitted with the mgcv package, and the significance of individual coefficients was evaluated using F-tests.

## 3. Results

### 3.1. Yearly Variations in BC Concentration

**Figure 2:**
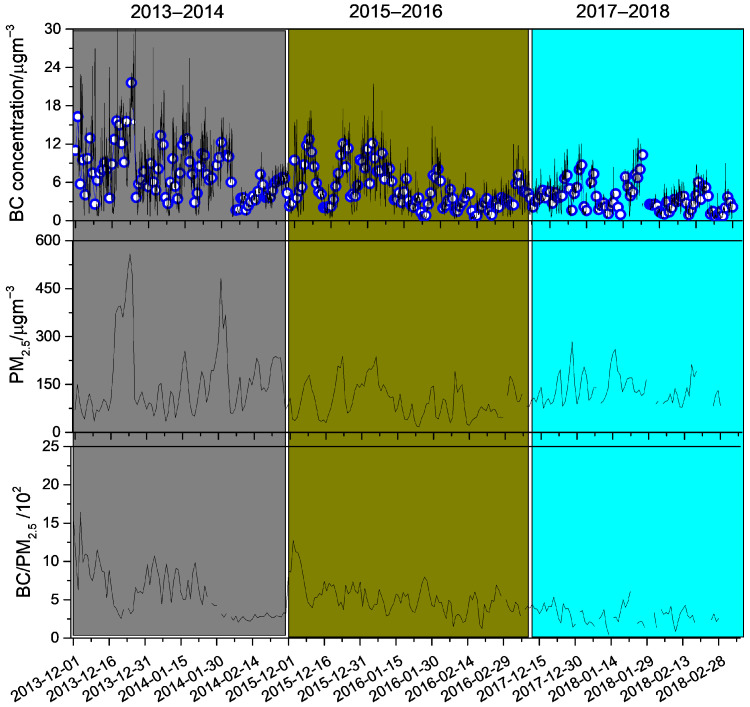
Temporal variations in 1 h black carbon (BC) concentrations, 24 h PM_2.5_ concentrations, and 24 h BC/PM_2.5_ ratios (blue circle represents 1 h BC concentration) in 2013–2014 (gray shadow), 2015–2016 (dark yellow shadow), and 2017–2018 (light blue shadow).

As presented in Figure 2, daily (24 h) mean BC concentrations were highest in winter 2013–2014 (7.5 ± 4.0 μg·m^−3^) and declined by a factor of 1.5–2 in 2015–2016 (5.2 ± 2.1 μg·m^−3^) and 2017–2018 (3.6 ± 1.1 μg·m^−3^). Across all winters, daily BC varied substantially (0.2 to 35.3 μg·m^−3^), indicating pronounced day-to-day fluctuations. Wintertime PM_2.5_ also showed strong variability, with the highest mean in 2013–2014 (162.4 ± 116.5 μg·m^−3^), followed by 2017–2018 (131.8 ± 45.7 μg·m^−3^) and 2015–2016 (96.4 ± 55.5 μg·m^−3^). Using daily means provides a consistent day-scale metric for interannual comparisons and, relative to sub-daily series, reduces short-lag serial dependence associated with diurnal cycling and short-term meteorological variability [39,40]. Consistent with the occurrence of shared pollution episodes, the daily PM_2.5_ and BC series were positively correlated in all three winters (Figure S2; r = 0.70 in 2013–2014, 0.80 in 2015–2016, and 0.60 in 2017–2018), with *p* < 0.001 for all periods. Because these are still time series influenced by multi-day stagnation, boundary-layer dynamics, and regional transport, we treat the correlation as a descriptive indicator of co-variation rather than definitive proof of co-emission or a single common source [41,42]. Additional constraints point to substantial shifts in dominant sources across winters: BC/PM_2.5_ ratios ranged from 0.012 to 0.164 during the sampling periods [26], with the smallest ratios (<0.05) mainly associated with residential solid-fuel combustion, whereas higher ratios (>0.1) were more frequent in 2013–2014, consistent with stronger contributions from industrial coal combustion and traffic emissions.

Table 1 indicates that the BC/CO ratios showed high variability during the sampling periods, ranging from 0.5 to 5.2. The average BC/CO ratio in this study appeared to be heavily influenced by diesel/gasoline vehicle and industrial emissions [25] and was determined to be comparable to measurements for urban plumes, such as those in Beijing [43], Shanghai [44], Guangzhou [45], Gwanjun [46], Bangkok [47], and Tokyo [48]. Moreover, the BC/CO ratios were considerably lower than those of biomass burning plumes. Studies on biomass combustion in different regions have reported greatly varying BC/CO ratios, which were relatively high for forest fires (28.5, [49]) and open burning of crop residue (8.9–9.4, [50]), reaching as low as 3.2 for crop residue burning over the North China Plain and in Central China [42]. Thus, the types of mass burned and combustion conditions affect BC/CO. As a central city in northwestern China, the heavy combustion of low-quality biomass fuels for heating during the cold winter leads to relatively high BC/CO over the Xi’an region.

### 3.2. Light-Absorbing Properties and Potential Source Regions of BC and BrC

**Figure 3:**
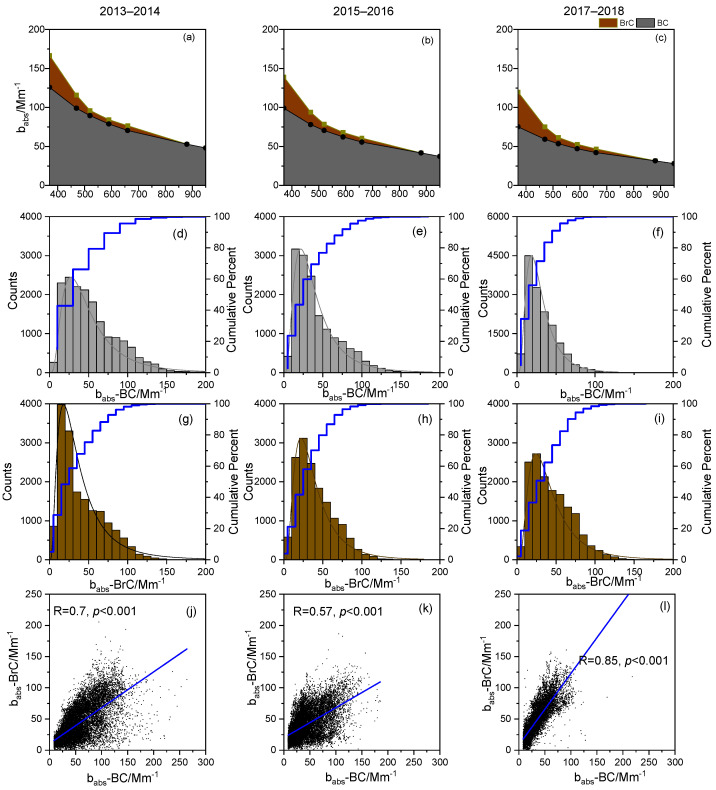
(**a**–**c**) Proportions of light absorptions of black carbon (BC) and brown carbon (BrC) to total light absorption (b_abs_) in different wavelengths. Frequency distributions of (**d**–**f**) *b*_abs_-BC and (**g**–**i**) *b*_abs-_BrC. (**j**–**l**) correlations between *b*_abs_-BC and *b*_abs-_BrC.

In Figure 3a–c, the yearly average b_abs_-BC was the dominant contributor to particulate absorption over the seven wavelengths and decreased in winter over Xi’an. Previous studies confirmed that the light absorption coefficient of BC at 880 nm is representative of actual BC absorption without BrC effects [42,51]. Similarly to the observed BC concentration, b_abs_-BC exhibited a decreasing trend from 60.0 Mm^−1^ during 2013–2014 to 42.4 Mm^−1^ during 2015–2016 and 32.1 Mm^−1^ during 2017–2018. As presented in other studies [22,23,29], the data of b_abs_ in the UV spectra (370 nm) (b_abs_-BrC) were selected to represent b_abs_ data for ambient BrC in this study. In contrast to the preceding results, the winter b_abs_-BrC value was high (41.7 Mm^−1^) during 2017–2018 but decreased to 37.9 Mm^−1^ during 2015–2016 and to 35.6 Mm^−1^ during 2013–2014. Similarly, the contribution of b_abs_-BrC to total b_abs_ at the 370 nm wavelength increased substantially in each winter period, accounting for approximately 15% during 2013–2014, 17% during 2015–2016, and as much as 40% during 2017–2018. These trends demonstrate that the influence of BrC on light absorption was substantially strengthened.

As shown in Figure 3d–f, the distribution of babs-BC in different wintertime exhibited distinct variations. The b_abs_-BC values had a flat and wide range (15–80 Mm^−1^) in 2013–2014, but in subsequent years, they were distributed in narrow ranges (10–30 Mm^−1^, >70%). Furthermore, only approximately 8% of the data exceeded 100 Mm^−1^ and 70 Mm^−1^ during 2015–2016 and 2017–2018 (Figure 3d–f), respectively, possibly because of changes in meteorological conditions and major contribution sources [24]. By contrast, winter b_abs-BrC_ exhibited an approximately unimodal distribution, with about 60% of values concentrated within 15–50 Mm^−1^ (Figure 3g–i) during the sampling periods, indicating a broadly consistent range of BrC absorption levels across winters. Moreover, the relationship between b_abs_-BrC and b_abs_-BC during these periods was demonstrated by the strong correlation observed during the winters of 2013–2014 and 2017–2018 and the weak correlation during that of 2015–2016 (0.57). Thus, we infer that BC and BrC are often co-emitted from common primary combustion sources, particularly incomplete residential coal and biomass burning, consistent with source-emission/combustion studies reporting the co-occurrence of BC and BrC absorption in emissions from combustion fuels, including mixed biomass and fossil-fuel combustion [23]. However, these findings also demonstrate that considerable amounts of BrC in 2015–2016 were produced from secondary rather than direct emissions (see Section 3.3).

**Figure 4:**
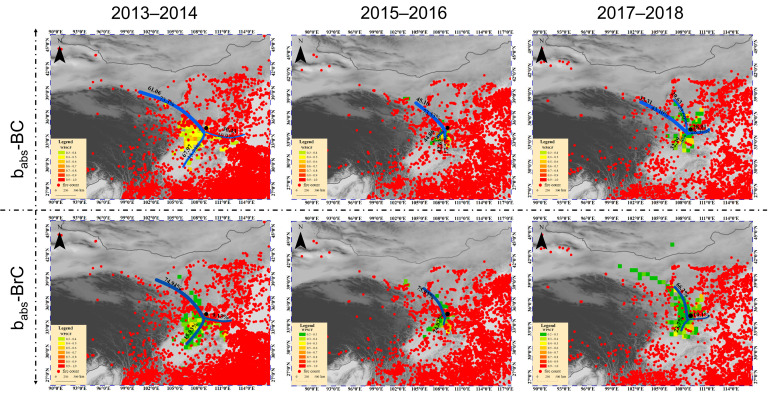
Cluster trajectories and 75th-percentile potential source contribution function probabilities of aethalometer-generated black carbon (BC) and brown carbon (BrC) light absorption (b_abs_-BC and b_abs_-BrC) reaching Xi’an during sampling periods (The red points represent fire points, and the blue lines represent pollution cluster trajectories).

Figure 4 illustrates the results of the cluster analysis and potential source regions with weighted PSCF values for higher concentrations (above the 75th percentile) of b_abs_-BC and b_abs_-BrC in Xi’an. For b_abs_-BC, the trajectories were grouped into three clusters during the entire sampling period. Cluster 1 from the northwestern direction was the largest (~73%), and the air masses associated with this cluster yielded the lowest PSCF values (<0.3) among the three clusters, indicating that air from Cluster 1 was less polluted. However, during 2013–2014 and 2015–2016, the other two polluted clusters (Clusters 2 and 3) of b_abs_-BC were derived from the south–southwestern direction (>26% of the wintertime trajectories), which had high PSCF values (0.4–0.7). Moreover, dense open fire points in the south–southeast were well matched with these clusters during these two winter periods (Figure S3), indicating that b_abs_-BC was largely affected by suburban area and local biomass combustion emissions [22,52]. By contrast, higher PSCF values (0.4–0.5) were mainly concentrated in clusters 2 and 3 during the winter of 2017–2018 in the north–northeast and southwest of Xi’an. These regions weakly corresponded to open fire points, possibly because of the intensive burning of fossil fuels. Similarly to b_abs_-BC, b_abs_-BrC was also heavily influenced by local emissions during the winter sampling periods. In addition, the b_abs_-BrC clusters from the north-northeast direction had high PSCF values (0.4–0.6) during 2017–2018. These emissions were transported from surrounding regions and provinces (e.g., Yulin and Shanxi province), where coal remains widely used in industrial and residential sectors that increase BrC levels [53,54].

### 3.3. Assess BC and BrC Sources

**Figure 5:**
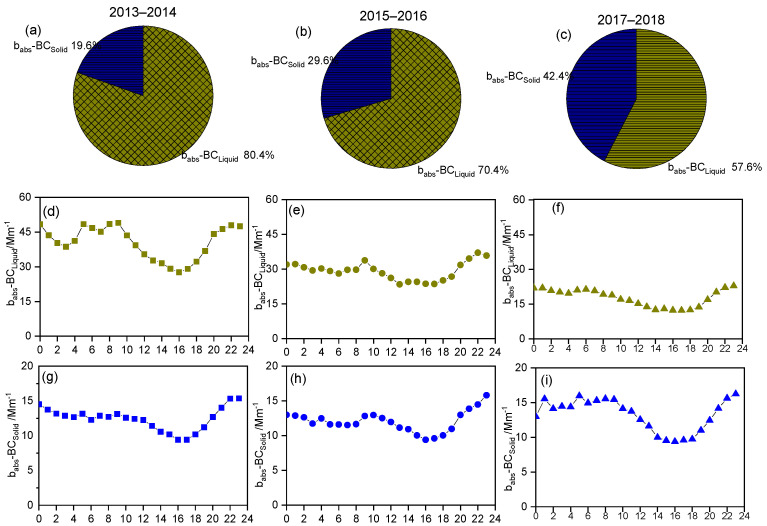
(**a**–**c**) Relative contributions of b_abs_-_Liquid_ and b_abs_-BC_Solid_. (**d**–**i**) Diurnal variations in b_abs_-_Liquid_ and b_abs_-BC_Solid_.

To investigate BC sources in depth, the relative contributions of liquid and solid sources to b_abs_-BC were apportioned using the aethalometer model, as presented in Figure 5a–c. The average concentration of b_abs_-_Liquid_ in Xi’an was 32.1 ± 15.1 Mm^−1^, approximately three times the value of b_abs_-BC_Solid_. In addition, b_abs_-_Liquid_ had a wider concentration range of 10.5–111.4 Mm^−1^, whereas b_abs_-BCS had a narrower range of 5–15 Mm^−1^, indicating that BC absorptions in Xi’an were significantly influenced by vehicle emissions and varied with traffic flow. In addition, higher b_abs_-_Liquid_ fractions of 80.4% and 70.4% were observed during 2013–2014 and 2015–2016, respectively, whereas solid sources contributed up to approximately 30% of BC absorption during 2017–2018, indicating that major BC pollution sources changed to biomass/coal combustion emissions. In terms of the diurnal cycles of b_abs_-_Liquid_ (Figure 5d–f), b_abs_-_Liquid_ exhibited a bimodal distribution with two obvious high points during morning 06:00 local standard time (LST) and evening (18:00 LST traffic rush hours [55,56]. Moreover, b_abs_-BC_Liquid_ did not decrease after the evening rush hour mainly because of the lowering of the local nocturnal boundary layer, allowing little space for pollutants to accumulate. Unlike other urban areas, the third peak of b_abs_-BC_Liquid_ emissions from diesel vehicles occurred at approximately midnight and in the early morning because diesel trucks are allowed to drive on the second-ring roads. These b_abs_-BC_Liquid_ peaks were higher than those of gasoline emissions, indicating the strong BC intensity of diesel emissions [57,58,59]. In contrast to that of b_abs_-_Liquid_, only one peak observed for b_abs_-BC_Solid_ persisted nearly throughout the night (18:00–06:00 LST), and minimum b_abs_-BC_Solid_ values were observed on all days. This indicates that these peaks were closely related to changes in household heating at night, thereby verifying that contributions of solid sources did not decrease substantially during these years.

**Figure 6:**
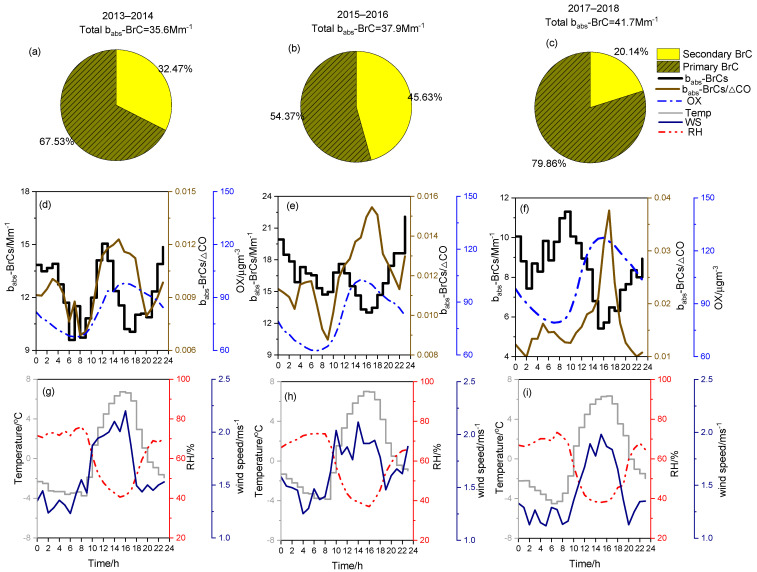
(**a**–**c**) Proportion of b_abs_-BrC_P_ and b_abs_-BrC_S_ in 3 years. (**d**–**i**) Temporal evolution of b_abs_-BrC_S_ in 3 years; diurnal variations (local time [LT]) of b_abs_-BrC_S_/ΔCO ratios (ΔCO = (1–1.25%) × measured CO values), relative humidity (RH), temperature, windspeed, and odd oxygen (OX = NO + O_3_) values.

Figure 6a–c illustrates the average quantities of b_abs_-BrC_P_ and b_abs_-BrC_S_ during all sampling periods. The b_abs_-BrC_P_ values were 24.0, 20.6, and 33.3 Mm^−1^ in the years of 2013–2014, 2015–2016, and 2017–2018, respectively. Moreover, high ratios of b_abs_-BrC_P_ to total BrC (54.4–80.0%) were observed, suggesting that primary emissions produced from household heating (coal and biomass combustion) play an important role in BrC contributions [60,61]. Relatively low contributions of b_abs_-BrC_S_ to BrC were observed during 2013–2014 and 2017–2018, but they reached 45.6% during 2015–2016 because of changes in energy structures and BrC formation mechanisms.

To explain BrC formation, this study investigated the diurnal cycles of S-BrC concentration, b_abs_-BrC_S_/ΔCO ratio, and odd-oxygen-mixing ratio (OX = NO_2_ + O_3_), as plotted in Figure 6d–f. The diurnal patterns of b_abs_-BrC_S_ were similar during 2013–2014 and 2015–2016 and peaked in midday 11:00–13:00 LST and at late night (22:00–02:00) LST. In addition, the b_abs_-BrC_S_/ΔCO ratio increased at the same time because of activated secondary formation. Combined with variable meteorological conditions, the b_abs_-BrC_S_ peak at noon possibly occurred because of photochemical reactions with high OX, whereas the late-night b_abs_-BrC_S_ peak could be attributed to aqueous reactions under high-RH conditions [29,62]. By contrast, completely inverse variations in b_abs_-BrC_S_ and b_abs_-BrC_S_/ΔCO were observed during 15:00–16:00 LST, indicating that the photochemical production rate of b_abs_-BrC_S_ was much lower than the photodegradation rate. Moreover, reduced b_abs_-BrC_S_ occurred at times with beneficial dispersion conditions and a relatively high boundary layer. During 2017–2018, the b_abs_-BrC_S_ values were completely different from those observed during the other two years. No substantial peak was observed at midday, whereas b_abs_-BrC_S_ retained high values after 20:00 LST for a relatively long time [63]. It was confirmed that the quantity of total chromophore observed in Xi’an in the winter of 2017 was poorly associated with secondary photochemical production of BrC. The high b_abs_-BC_S_ proportion in our study suggests that secondary BrC is mainly produced from aqueous reactions from fossil fuel burning and contains high levels of light-absorbing chromophores that could significantly affect the light-absorbing abilities of organic aerosols in the atmosphere [64].

### 3.4. Quantifying the Influence of Local Sources and Meteorology on BC

After the heavy pollution event in the winter of 2013, the Xi’an government implemented restrictions on vehicle license plate numbers and coal-to-gas conversion. Figure 1 indicates that BC emissions in Xi’an decreased by 32% and 50% from 2013 to 2014 and 2015–2016, as well as 2017–2018, respectively. Thus, GAMs for ln(b_abs_-_Liquid_) and ln(b_abs_-BC_Solid_) before (2013–2014) and after (2015–2018) the control period were applied to further assess the key emission effects from all observed gaseous pollutants and meteorological conditions. The R2 value calculated for the GAMs ranged from 0.5 to 0.7, indicating that the GAMs could explain more than 50% and 70% of the ln(b_abs_-_Liquid_) and ln(b_abs_-BC_Solid_) variance during 2013–2014 and 2015–2018, respectively.

**Figure 7:**
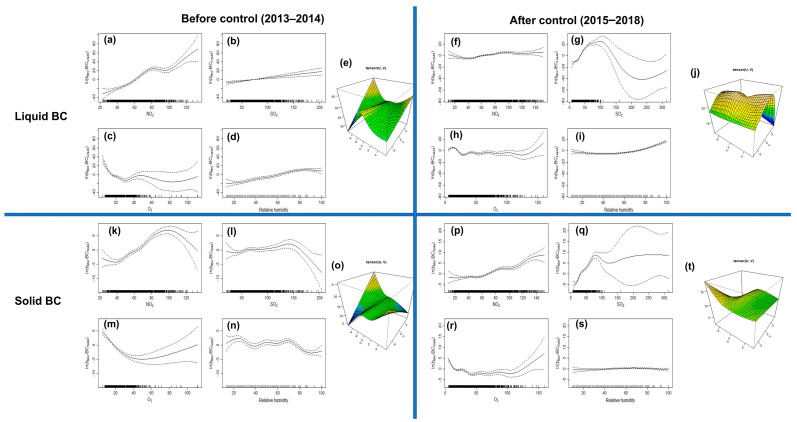
Fitted components of the ln(b_abs_-BC*_Liquid_*) and ln(b_abs_-BC*_Solid_*) model. Smooth functions are shown for (**a**,**f**,**k**,**p**) NO_2_, (**b**,**g**,**l**,**q**) SO_2_, (**c**,**h**,**m**,**r**) O_3_, (**d**,**i**,**n**,**s**) RH, (**e**,**j**,**o**,**t**) wind direction and windspeed. Note: The horizontal and vertical axes of the one-dimensional graph separately indicate the independent variable and its smooth function (tensor function for wind speed and wind direction); the dashed line areas show the estimate of the 95% confidence intervals, and the vertical dashes at the bottom of the plot illustrate locations where data for the plotted covariate informs the model estimates.

During 2013–2014, NO_2_ and RH accounted for 26.3% and 8.2% of the variance in b_abs_-_Liquid_ levels at the 99% confidence level, respectively, and both increased linearly with ln(b_abs_-_Liquid_), indicating that high RH (50–70%) under relatively stable meteorological conditions (wind speed < 2 m·s^−1^) could accelerate the condensation, nucleation, and growth of vehicle emissions [65,66], leading to the accumulation of b_abs_-_Liquid_. After the implementation of restrictions on vehicle license plate numbers, the contribution of NO_2_ decreased by 4.1 times during 2015–2018, demonstrating the high effectiveness of the restrictions in reducing BC concentrations. In contrast to those observed during 2013–2014, high SO_2_ levels explained 56.6% of ln(b_abs_-_Liquid_), confirming that increased diesel truck emissions at night contributed the most to b_abs_-_Liquid_ levels during 2015–2018. Unlike the increasing variables, the variance in O_3_ decreased linearly with increased ln(b_abs_-_Liquid_), indicating that b_abs_-_Liquid_ decreases rapidly with high oxidant levels because O_3_ can catalyze the photochemical reaction of BC, such that it is mostly consumed. In addition, these decreasing effects occurred only in low-O_3_ ranges (<25 μg·m^−3^), whereas O_3_ deviance became large and sparse when its value exceeded 50 μg·m^−3^. Other meteorological conditions, such as wind speed and direction, contributed less to the deviance of b_abs_-_Liquid_ with low variance ranges (<2%). For ln(b_abs_-BC_Solid_), combined NO_2_ and SO_2_ explained 49.3% and 75.4% of ln(b_abs_-BC_Solid_) in 2013–2014 and 2015–2018, respectively. Tian et al. [67] demonstrated that inefficient combustion of raw coal for heating can produce large amounts of NOx and SO_2_. Thus, winter heating emissions from coal combustion constituted a substantial fraction of b_abs_-BC_Solid_. However, Figure 7 indicates that increased SO_2_ concentrations during 2015–2018 exhibited a significantly linear relationship with ln(b_abs_-BC_Solid_) (*p* < 0.001, F-test, Table 2) compared with those during 2013–2014. This indicates that improving coal combustion technology in winter remains an effective means of reducing the production of b_abs_-BC_Solid_. Similar to b_abs_-_Liquid_, RH affected b_abs_-BC_Solid_ accumulation more during 2013–2014 than during 2015–2018. By contrast, the combination of wind speed and wind direction appeared to be an increasing contributor (>10%) to the transportation of polluted air in the southern (V < 0) and western (U < 0) directions, leading to high ln(b_abs_-BC_Solid_) values during 2013–2014. Similarly, the PSCF and cluster analyses highlighted the influence of biomass combustion.

### 3.5. Source Apportionment of BrC

**Figure 8:**
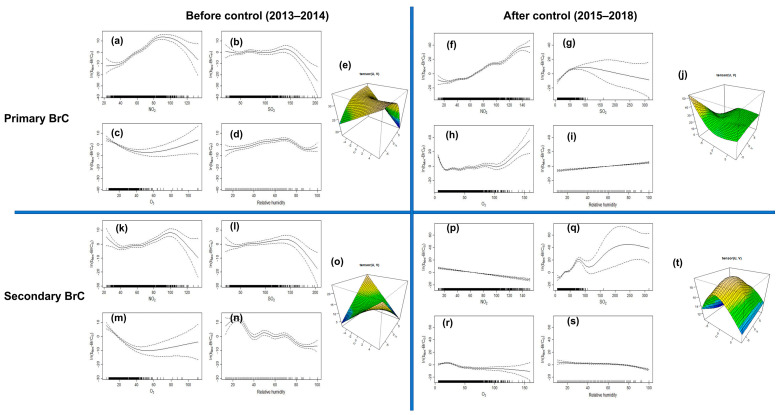
Fitted components of the ln(b_abs_-BrC_P_) and ln(b_abs_-BrC_S_) model. Smooth functions are shown for (**a**,**f**,**k**,**p**) NO_2_, (**b**,**g**,**l**,**q**) SO_2_, (**c**,**h**,**m**,**r**) O_3_, (**d**,**i**,**n**,**s**) RH, (**e**,**j**,**o**,**t**) wind direction and windspeed. Note: The horizontal and vertical axes of the one-dimensional graph separately indicate the independent variable and its smooth function (tensor function for wind speed and wind direction); the dashed line areas show the estimate of the 95% confidence intervals, and the vertical dashes at the bottom of the plot illustrate locations where data for the plotted covariate informs the model estimates.

To quantify BrC sources, GAMs were created for ln(b_abs_-BrC_P_) and ln(b_abs_-BrC_S_). Gaseous pollutants and meteorological parameters in the GAMs could explain 41–60% of ln(b_abs_-BrC_P_) variance but only 20–30% of ln(b_abs_-BrC_S_) (Table 2). Unlike those of BC, similar BrC values were observed during the sampling periods. High NO_2_ and SO_2_ concentration levels contributed considerably (>50%) to ln(b_abs_-BrC_P_) before and after the control period, confirming the fossil fuel combustion influences. As supported, Lei et al. [19] highlighted that 35% of primary BrC in winter was derived from traffic emissions and coal combustion. Similarly to BC, RH was a major meteorological parameter for high ln(b_abs_-BrC_P_) during 2013–2014. In addition, approximately 5% of the variability in ln(b_abs_-BrC_P_) was related to wind speed and direction during 2013–2014, indicating that high wind speed from the west–southwestern direction was conducive to primary BrC diffusion. Regarding O_3_ effects (Figure 8), a similarly linear decreasing trend showed photodegradation of b_abs_-BrC_P_ was significant in Xi’an winters. The low R2 value for ln(b_abs_-BrC_S_) calculated in the GAMs suggests that a substantial portion of ln(b_abs_-BrC_S_) could not be explained by the parameters in the current model. More parameters must be considered for GAMs to quantify b_abs_-BrC_S_ in future work.

## 4. Conclusions

This study investigated the concentrations and optical properties of BC and BrC during three winter periods from 2013 to 2018. Optical parameters were used along with PSCF analysis, and the results reveal that both BC and primary BrC were typically affected by direct local emissions and the transportation of polluted air masses from surrounding biomass-burning areas or coal-dominated regions. A high secondary BrC ratio was also observed in winter, indicating that aqueous transforming reactions were dominant sources of BrC. Moreover, a GAM statistical methodology was used to evaluate the contributions of gaseous pollutants and meteorological conditions before (2013–2014) and after (2015–2018) air pollution control policies. Most variations in b_abs_-BC derived from liquid sources could be explained by NO during 2013–2014 and by SO during 2015–2018, indicating that restrictions on vehicle license plate numbers were effective in reducing gasoline vehicle influences and had a limited effect on diesel emissions. The combination of SO and NO concentrations emitted from residential coal combustion for heating explained the significant increase in solid b_abs_-BC and primary b_abs_-BrC. In addition, meteorological variables exhibited multilinear relationships with b_abs_-BC and b_abs_-BrC in different years, indicating that the meteorological variables had considerable effects on the accumulation and dispersion of BC and BrC emissions across the studied years.

## Figures and Tables

**Table 1:** Comparison of BC and CO mass concentrations and BC/CO ratios with other urban observations and near-source measurements.

Urban Areas					
Region	Sampling Periods	BC/μg·m^−3^	CO/mg·m^−3^	BC/CO (10^−3^)	Reference
Xi’an	2013–2014 December–February	7.4	2	0.9	This study
	2015–2016 December–February	4.9	2.5	0.5–3.7	
	2017–2018 December–February	3.7	2	0.8–4.0	
Beijing	2005–2006 winter	6.7	1.6	4.2	[43]
Guangzhou	2006 July	4.7	1.0	4.7	[45]
Shanghai	2005	2.4–5.5	0.2–2.5	3.7	[44]
Tokyo	2003–2005	1.8	0.4	4.6	[48]
Nagoya	2003 March	/	/	5.0	
Bangkok	2007–2008 dry season	/	/	6.3	[47]
	2007–2008 wet season	/	/	7.8	
Gwanjun (traffic-dominated)	2001 March–April	2.7–3.8	0.4–0.7	3.7–5.4	[46]
North China Plain–Central China	2017 January	3.9–8.5	/	3.2–12.8	[42]
Near-sources					
Industry				5.7	[25]
Traffic				4.1	
Biomass burning—forest fire				28.5	[49]
Biomass burning—crop residue				8.9–9.4	[50]

**Table 2:** F-statistics and *p*-values for the F-tests of each coefficient of the ln(*PM*_2.5_) and ln(*BC*) GAMs.

	**Variance**	**Before Control (2013–2014)**						**After Control (2015–2018)**					
BC		ln(*b_abs_-BC_Liquid_*)			ln(*b_abs_-BC_Solid_*)			ln(*b_abs_-BC_Liuqid_*)			ln(*b_abs_-BC_Solid_*)		
		F-statistic	% of variance	*p*	F-statistic	% of variance	*p*	F-statistic	% of variance	*p*	F-statistic	% of variance	*p*
	s(NO)	51.2	26.3	<0.001	35.6	26.7	<0.001	16.2	6.4	<0.001	39.2	10.4	<0.001
	s(O_3)_	15.3	56.5	<0.001	42.6	9.7	<0.001	24.2	33.6	<0.001	24.6	21.7	<0.001
	s(SO)	19.9	0.0	<0.001	2.9	22.6	<0.001	133.3	56.6	<0.001	83.7	65.0	<0.001
	s(RH)	36.5	8.2	<0.001	11.7	30.5	<0.001	87.5	1.5	<0.001	2.5	0.8	0.060
	te(U,V)	1.6	0.0	0.165	1.8	10.4	0.101	1.7	1.9	0.067	2.9	2.0	0.002
BrC		ln(*b_abs_-BrC_P_*)			ln(*b_abs_-BrC_S_*)			ln(*b_abs_-BrC_P_*)			ln(*b_abs_-BrC_S_*)		
		F-statistic	% of variance	*p*	F-statistic	% of variance	*p*	F-statistic	% of variance	*p*	F-statistic	% of variance	*p*
	s(NO)	41.9	35.5	<0.001	7.9	28.3	<0.001	153.2	35.3	<0.001	147.6	0.0	<0.001
	s(O_3)_	18.9	9.5	<0.001	36.2	9.1	<0.001	51.9	43.8	<0.001	19.4	5.8	<0.001
	s(SO)	3.5	30.2	<0.001	3.1	16.4	0.003	62.2	15.6	<0.001	6.0	91.6	<0.001
	s(RH)	138	22.5	<0.001	36.4	46.2	<0.001	101.4	0.0	<0.001	27.0	1.6	<0.001
	te(U,V)	1.3	2.3	0.262	2.2	0.0	0.087	1.6	5.2	0.081	3.0	1.0	0.003

## Data Availability

The data presented in this study are available on request from the corresponding author.

## Supplementary Materials

The following supporting information can be downloaded at: https://www.mdpi.com/article/10.3390/toxics14010093/s1. Figure S1: R2 from regression between b_abs_-BrC_S_ versus BC and assumed (b_abs_)/BC)_P_ ratio in three years; Figure S2: Correlations of PM_2.5_ and BC concentrations; Figure S3: Fire counts during sampling periods.
