# Supplementary material for: Insight into the Formation of Winter Black Carbon and Brown Carbon over Xi’an in Northwestern China
Source: Toxics. 2026 Jan 20;14(1):93. doi: 10.3390/toxics14010093 (PMC12846155; doi:10.3390/toxics14010093)
Supplement: Supplementary file 1 [file toxics-14-00093-s001.zip › toxics-4035741-supplementary.pdf]

Article

Insight into the Formation of Winter Black Carbon and Brown Carbon over Xi'an in Northwestern China

Dan Li [1,2], Qian Zhang [3,*], Ziqi Meng [3], Hongmei Xu [1], Peng Wei [4], Yu Wang [3] and Zhenxing Shen [1]

[1] Department of Environmental Science and Engineering, Xi'an Jiaotong University, Xi'an 710049, China; lidan@ieecas.cn (D.L.); xuhongmei@xjtu.edu.cn (H.X.); zxshen@mail.xjtu.edu.cn (Z.S.)
[2] State Key Laboratory of Loess Science, Institute of Earth Environment, Chinese Academy of Sciences, Xi'an 710061, China
[3] Key Laboratory of Northwest Resource, Environment and Ecology, MOE, Xi'an University of Architecture and Technology, Xi'an 710055, China; mengziqi@xauat.edu.cn (Z.M.); 15943255731@163.com (Y.W.)
[4] Division of Environment and Sustainability, The Hong Kong University of Science and Technology, Hong Kong, China; pengwei@sdnu.edu.cn
* Correspondence: zhangqian2018@xauat.edu.cn

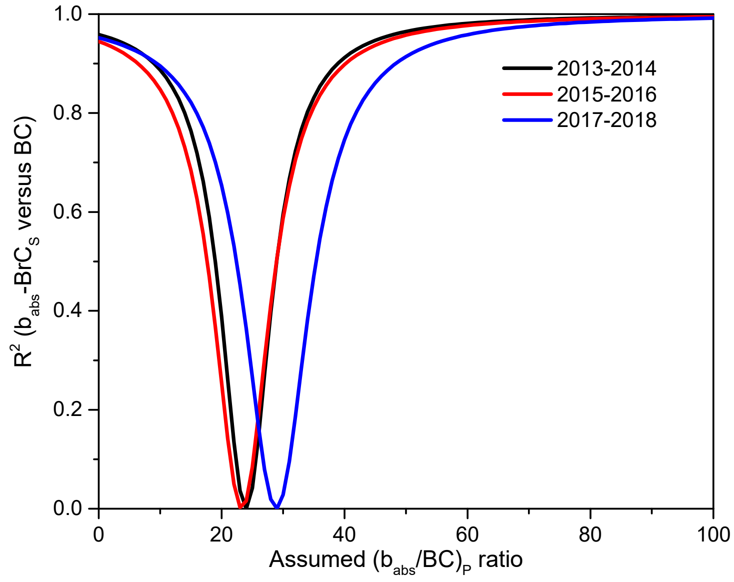

Figure S1. R^2 from regression between b_{abs}-BrC_S versus BC and assumed $(b_{abs})/BC)_P$ ratio in three years.

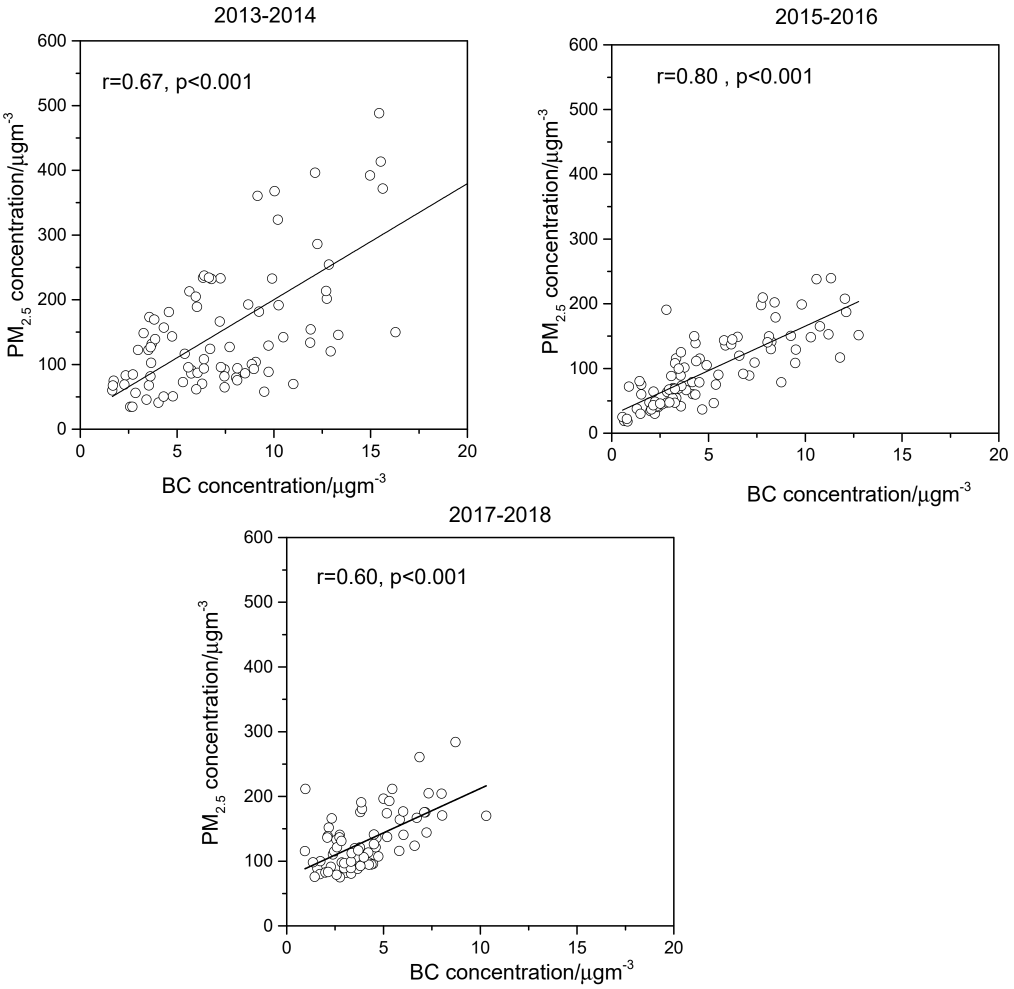

Figure S2. Correlations of $PM_{2.5}$ and BC concentrations.

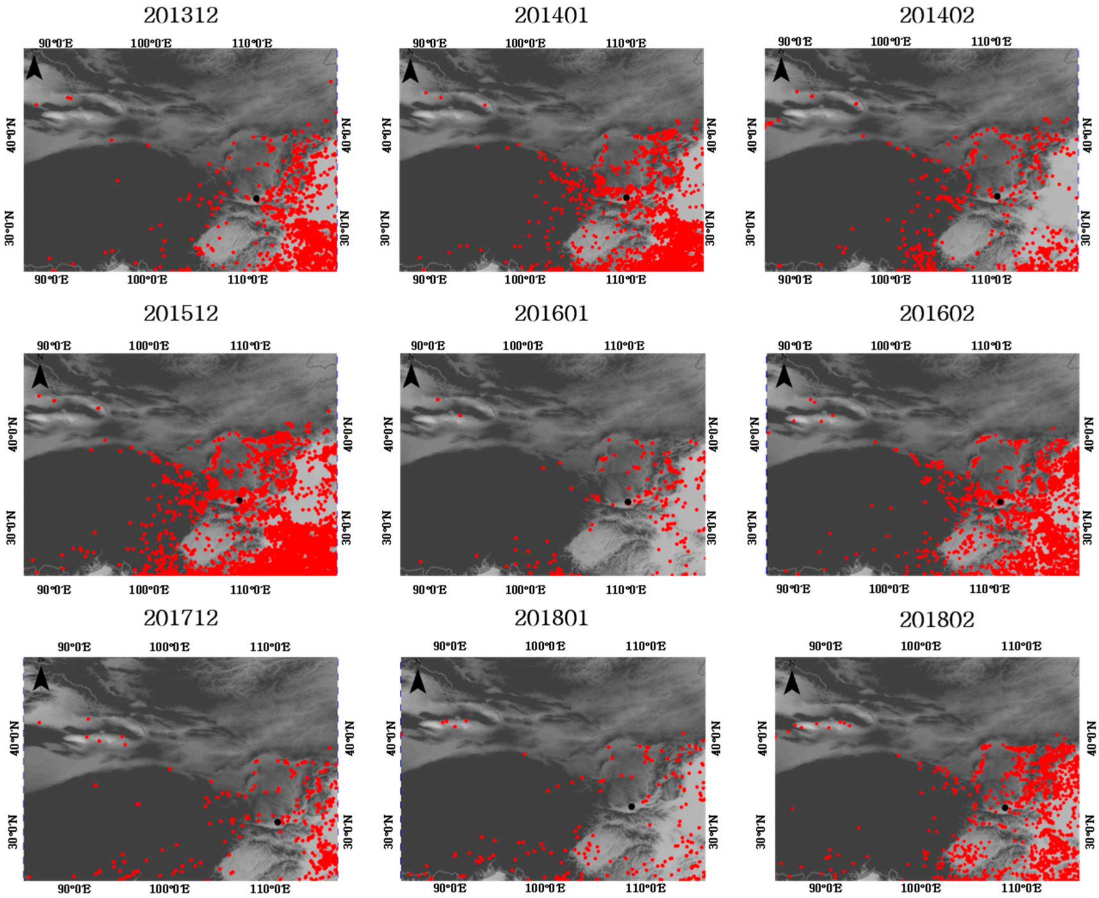

Figure S3. Fire counts during sampling periods.
